# Inhibition of *TREM-1* and *Dectin-1* Alleviates the Severity of Fungal Keratitis by Modulating Innate Immune Responses

**DOI:** 10.1371/journal.pone.0150114

**Published:** 2016-03-10

**Authors:** Jing Zhong, Weilan Huang, Qiuchan Deng, Minhao Wu, Huaili Jiang, Xiaolei Lin, Yifang Sun, Xi Huang, Jin Yuan

**Affiliations:** 1 State Key Laboratory of Ophthalmology, Zhongshan Ophthalmic Centre, Zhongshan School of Medicine, SunYat-sen University, Guangzhou, 510064, China; 2 Department of Immunology, Institute of Human virology, Zhongshan School of Medicine, Sun Yat-sen University, Guangzhou, 510080, China; 3 Key Laboratory of Tropical Diseases Control (Sun Yat-sen University), Ministry of Education, Guangzhou, 510080, China; 4 Department of Otolaryngology Head and Neck Surgery, Sun Yat-sen Memorial Hospital, Sun Yat-sen University, Guangzhou, China; University of Crete, GREECE

## Abstract

**Purpose:**

To explore the possibility that inhibiting triggering receptor expressed on myeloid cells-1 (TREM-1) and Dendritic cell-associated C-type lectin-1(Dectin-1) could modulate the innate immune response and alleviate the severity of corneal fungal keratitis.

**Method:**

TREM-1 and Dectin-1 expression was detected in fungus-infected human corneal specimens by real-time PCR. C57BL/6 (B6) mice were injected with *Aspergillus fumigatus* and divided into 4 groups that received subconjunctival injections of PBS and IgG as a control (group I), mTREM-1/IgG fusion protein (group II), the soluble β-glucan antagonist laminarin (group III), or mTREM-1/Fc and laminarin (group IV). Corneal virulence was evaluated based on clinical scores. TREM-1 and Dectin-1 mRNA levels were assayed using real-time PCR. The distribution patterns of TREM-1, Dectin-1 and cellular infiltrates in fungus-infected corneas were examined by immunohistochemistry. Moreover, changes in T Helper Type1 (Th1)-/ T Helper Type1 (Th2)- type cytokines and proinflammatory cytokines were measured.

**Results:**

The expression of TREM-1 and Dectin-1 increased significantly and correlated positively with the progression of fungal keratitis. Most infiltrated cells were neutrophils and secondarily macrophages in infected cornea. The clinical scores decreased after interfering with TREM-1 and Dectin-1 expression in infected mouse corneas. Levels of Th1-type cytokines including interleukin-12 (IL-12), IL-18 and interferon-γ (IFN-γ) were decreased in the cornea, while the levels of Th2-type cytokines, including IL-4, IL-5 and IL-10, showed obvious increases.

**Conclusion:**

TREM-1 and Dectin-1 function concurrently in the corneal innate immune response by regulating inflammatory cytokine expression in fungal keratitis. Inhibition of TREM-1 and Dectin-1 can alleviate the severity of corneal damage by downregulating the excessive inflammatory response.

## Introduction

Fungal keratitis is a severe, sight-threatening ocular disease because of trauma [1], the prevalence of contact lens use [2], corticosteroid abuse and ocular surgery [1]. Since its diagnosis is difficult, the availability of effective and specific antifungal agents is limited and its clinical outcome is poor, fungal keratitis is still a great challenge in ophthalmologic clinic [3]. Furthermore, even received an accurate diagnosis and appropriate treatment, 20% of fungal keratitis patients may suffer corneal perforation [4], which may be attributed to secondary corneal damage induced by excessive inflammatory responses.

The most common causative agents of fungal keratitis are *Candida* and *Aspergillus*. [5] Once fungi invade the corneal stroma, host innate immune cells recognize pathogens with pattern-recognition receptors (PRRs), especially C-type lectin receptors (CLRs). CLRs including mannose receptor (MR), Dectin-1 and Dectin-2, comprise transmembrane and soluble receptors that share a carbohydrate-recognition domain [6]. Recent findings have demonstrated that Dectin-1 is clearly expressed in the cornea and functions to detect invading fungi [7]. Dectin-1 can specifically recognizes β-glucan [8] which is a major conserved structural component of fungal cell walls. Activation of Dectin-1 signaling initiates a variety of inflammatory events, such as cellular maturation, the respiratory burst, the development of Th17 responses, as well as the production of inflammatory cytokines, such as IL-17A, TNF-α, IL-6, IL-1β and MIP-2.

In fungal keratitis, the clinical prognosis is largely determined by both pathogenic virulence and host immune response[9]. PRRs-mediated inflammatory response enhances clearance of fungi and promote tissue repair. However, if unchecked, prolonged over-reactive host immune response may amplify the inflammation, and lead to tissue injury and corneal perforation[10]. Therefore, precise regulatory mechanisms are required to modulate the inflammatory response in fungal keratitis.

Triggering receptors expressed on myeloid cells (TREMs) are identified as a new family of receptors that regulate both innate and adaptive immune responses to infection[10, 11]. Recent studies demonstrated that the expression of TREM-1 was strongly up-regulated in neutrophils and macrophages by lipopolysaccharide (LPS) and bacteria such as *P*. *aeruginosa*[10]. Inhibition of TREM-1 protects mice against LPS-induced septic shock and microbial sepsis caused by live *E*. *coli* [12]. In addition, it has been reported that TREM-1 can modulate immune responses to *P*. *aeruginosa* keratitis. Blocking TREM-1 with soluble mTREM-1/IgG fusion protein decreases Th1 response while enhances Th2 response, thus protects cornea from perforation[10]. However, the role of TREM-1 in fungal keratitis is largely unknown.

Here, we investigate the expressions and functions of TREM-1 and Dectin-1 in fungal keratitis. Our data reveal that both TREM-1 and Dectin-1 are significantly enhanced in either human or mouse corneas, which are infiltrated mainly by neutrophils and macrophages after fungal infection, and amplifies corneal inflammation by modulating Th1/Th2 immune responses. This study suggests that TREM-1 and Dectin-1 may have potential applications as targets for therapeutic intervention in fungal keratitis.

## Materials and Methods

### Patients and Tissue Specimens

Patient consent and approval from the Institutional Research Ethics Committee were obtained before these clinical samples were used for research purposes. All research with human subjects adhered to the tenets of the Declaration of Helsinki. Written informed consent was obtained from the participants or their guardians before the study, which conforms to the tenets of the Declaration of Helsinki. This study was approved by the Institutional Review Board of the Zhongshan Ophthalmic Center (approval ID: 2012KYNL017). Fungal keratitis patients who were treated at the Zhongshan Ophthalmic Center (Sun Yat-sen University, Guangzhou, China) from August 2012 to January 2014 were included in the study. The inclusion criterion was clinically diagnosed fungal keratitis that was experimentally confirmed by microbial culture of corneal scrapes, and the microbial culture revealed that they included 8 samples of Fusarium, 6 samples of Aspergillus fumigatus, and 6 samples of Candida albicans. Based on the infection time and severity of the corneal ulcer, the enrolled patients were divided into two groups. Patients in the early stage group had corneal infiltration limited to part of the cornea without hypopyon with a disease course lasting less than two weeks (7 males and 3 females, 23–71 years old). Patients in the late stage group had an infection lasting more than two weeks with serious corneal infiltration extending throughout the entire cornea (4 males and 6 females, 35–70 years old). These patients received corneal transplantation, and infected corneas were collected and analyzed using real-time polymerase chain reaction (PCR) and hematoxylin and eosin staining (H&E). Normal corneal tissues provided by an eye bank served as the control group. Donor corneas were confirmed to be free of any detectable prior pathologic conditions and were stored in a special storage reagent at a low temperature to maintain biological activity.

### Ocular Infection

The animal experiments complied with the Association for Research in Vision and Ophthalmology Statement for the Use of Animals in Ophthalmic and Vision Research. The research protocol was also approved by the Animal Care Committee of the Zhongshan Ophthalmic Center at Sun Yat-sen University (Guangzhou, China) (approval ID: 2013027). Eight-week-old female C57BL/6 (B6) mice were purchased from the Animal Supply Center of Sun Yat-sen University, Zhongshan School of Medicine, then the mice were anesthetized intraperitoneally with xylazine (1.9 mg/ml) and ketamine (37.5 mg/ml) and every effort was made to minimize suffering, and were placed beneath a stereoscopic microscope with 20× magnification. The right eye of each mouse was punctured to form a tunnel in the corneal stroma using a 30-gauge needle. Then, a 33-gauge Hamilton syringe was inserted through the tunnel, and 2 μl of a 1×10^5^ conidia (*Aspergillus fumigatus* strain no. 3.0772, China General Microbiological Culture Collection Center, Beijing, China) solution was injected into the corneal stroma[13]. The eyelids were rubbed together for several seconds to distribute the inoculum evenly over the corneal surface.

### Clinical Scoring

For the determination of clinical scores at 1, 3 and 5 days post-infection (p.i.), all infected corneas were photographed to illustrate the disease progression. Ocular disease was graded using clinical scores ranging from 0 to 12 according to the scoring system developed by Wu[13]. A total score of 5 or less indicated mild keratitis, 6 to 9 represented moderate keratitis and 9 to 12 indicated severe keratitis.

### Inhibition of TREM-1 and Dectin-1

B6 mice successfully infected with fungi were divided into 4 groups. Mice in group I had been given a subconjunctival injection of IgG and PBS (1 μg in 5 μl of PBS per cornea) (R&D Systems, Minneapolis, MN) into the right eyes before infection (n = 5/group/time) as a control. Then, on each day p.i., each mouse was injected i.p. with an additional 10 μg of IgG protein. Mice in group II, were given a subconjunctival injection of mTREM-1/IgG fusion protein (1 μg in 5 μl of PBS per cornea) (R&D Systems, Minneapolis, MN) into the right eyes before infection (n = 5/group/time), which competes with TREM-1 to bind with TREM-1 ligand[10]. Then, on each day p.i., each mouse was injected i.p. with an additional 10 μg of mTREM-1/IgG fusion protein. Mice in group III, were given a subconjunctival injection of the soluble β-glucan antagonist laminarin (1 μg in 5 μl of PBS per cornea) (Sigma-Aldrich, Germany) into the right eyes before infection (n = 5/group/time), which can specifically inhibit Dectin-1 expression [14, 15]. Then, on each day p.i., each mouse was injected i.p. with an additional 1 ml of laminarin (200 μg in 1 ml of PBS per mouse). Mice in group IV were given a subconjunctival injection of mTREM-1/Fc combined with laminarin. Then, on each day p.i., each mouse was injected i.p. with an additional 10 μg of mTREM-1/IgG fusion protein and an additional 1 ml of laminarin.

All mice were scored daily for corneal disease. The mice were sacrificed on days 1 and 5 p.i., and the eyes were then enucleated and processed for histological examination and PCR analysis. The animals were treated in compliance with the Association for Research in Vision and Ophthalmology (ARVO) Statement for the Use of Animals in Ophthalmic and Vision Research.

### Immunohistochemistry

Paraffin-embedded 4-μm sections of the infected corneas were deparaffinized with xylene and ethanol gradient methods and incubated for 10 min at room temperature with 3% freshly prepared hydrogen peroxide solution to block endogenous peroxidase activity. Antigen retrieval was performed via 3 cycles of microwaving slides submerged in citrate buffer (sodium citrate dihydrate, 2.94 g/L, pH 6.0) for 5 min at 50% power. The slides were then washed with PBS with 0.05% Tween-20(PBS-T), blocked with 5% horse serum for 30 min and incubated with rat monoclonal antibodies specific for mouse and human TREM-1 and Dectin-1 (R&D Systems, Minneapolis, MN),Macrophage(Abcam, Cambridge,UK) and Neutrophils which are identified by Gr-1 Ab (Thermo Scientific, West Palm Beach, FL). All primary antibodies were diluted 1:50 in PBS-T and incubated overnight at 4°C. The controls were treated similarly, although the primary antibody was replaced with isotype-matched IgG. The sections were then washed, incubated for 1 h at room temperature with secondary anti-rat antibodies (ImmPRESS reagent kit; Vector labs), washed with PBS and incubated with Black Alkaline Phosphatase Substrate (Vector labs, SK-5200) in the dark for 10–20 min. The reaction was terminated with double-distilled H_2_O, and the sections were counterstained with hematoxylin. The sections were then dehydrated in ethanol gradients, dipped in xylene and dried overnight before examination. All sections were visualized with a Zeiss microscope (Zeiss microscope AXIO Imager A1; Carl Zeiss, Inc, Oberkochen, Germany).

### Real-time PCR

Total RNA was isolated from individual corneas for analysis using TRIzol (Invitrogen, Carlsbad, CA) according to the manufacturer’s recommendations and was quantitated using NanoDrop 2000C spectrophotometers (Thermo Scientific, West Palm Beach, FL). One microgram of total RNA was reverse-transcribed to produce cDNA, and the cDNA was amplified using SYBR Green Master Mix (Bio-Rad, Hercules, CA) as suggested by the manufacturer. Primers for mouse IFN-γ, IL-4 and IL-5 were purchased from SABiosciences (Frederick, MD), and other primer sequences are listed in Table 1. Quantitative real-time PCR was performed using the CFX96 Real-Time PCR System (Bio-Rad, Hercules, CA). Relative gene expression levels were calculated after normalization to internal control β-actin.

**Table 1:** Nucleotide Sequences of the Specific Primers Used for PCR Amplification.

Gene	Primer Sequence (5'-3')	
mβ-actin	GAT TAC TGC TCT GGC TCC TAG C;	F
	GAC TCA TCG TAC TCC TGC TTG C	R
mTREM-1	TCC TAT TAC AAG GCT GAC AGA GCG TC;	F
	AAG ACC AGG AGA GGA AAC AAC CGC	R
mDectin-1	GACCCAAGCTACTTCCTC;	F
	GCAGCACCTTTGTCATACT	R
mIL-12	GGT CAC ACT GGA CCA AAG GGA CTA TG;	F
	ATT CTG CTG CCG TGC TTC CAA C	R
mIL-18	GCC TGT GTT CGA GGA TAT GAC TGA;	F
	TTC ACA GAG AGG GTC ACA GCC A	R
mIL-10	AGC TGG ACA ACA TAC TGC TAA CCG AC;	F
	CTT GAT TTC TGG GCC ATG CTT CTC TG	R
mIL-17A	TCAGCGTGTCCAAACACTGAG;	F
	CGCCAAGGGAGTTAAAGACTT	R
hβ-actin	GCT CCT CCT GAG CGC AAG;	F
	CAT CTG CTG GAA GGT GGA CA	R
hTREM-1	AGT CCC CAG GAT CAT ACT AGA AGA C;	F
	AGG CTG GTA GAT CAC ACA CTG ATA C	R
hDectin-1	GCT TAA TTG GAA AGA AGA GAA GA;	F
	GAT TAA AGG GAA ACA GGT ATC TT	R

### Enzyme-linked immunosorbent assay (ELISA)

Cytokine protein levels were selectively tested using ELISA kits (R&D Systems, Minneapolis, MN). Corneal samples were individually collected (n = 5/group/time) at 1 and 5 days p.i. from the different groups. Corneas were homogenized in 0.5 ml of PBS containing 0.1% Tween-20. All samples were centrifuged at 13,000 rpm for 5 min, and supernatants were collected. An aliquot of each supernatant was assayed in duplicate for TNF-α and IL-6 expression In accordance with the manufacturer’s instructions. The reported sensitivities of these assays are 5.1 pg/ml for TNF-α and 1.3 to 1.8 pg/ml for IL-6.

### Statistical Analysis

Differences in the clinical scores of the four groups at the corresponding time points were determined using the Mann-Whitney U test. An unpaired, two-tailed Student’s *t* test was used to determine the statistical significance of the other assays. The data were considered statistically significant at *P*<0.05.

## Results

### Expressions of TREM-1 and Dectin-1 in fungus-infected human corneas

**Fig 1:**
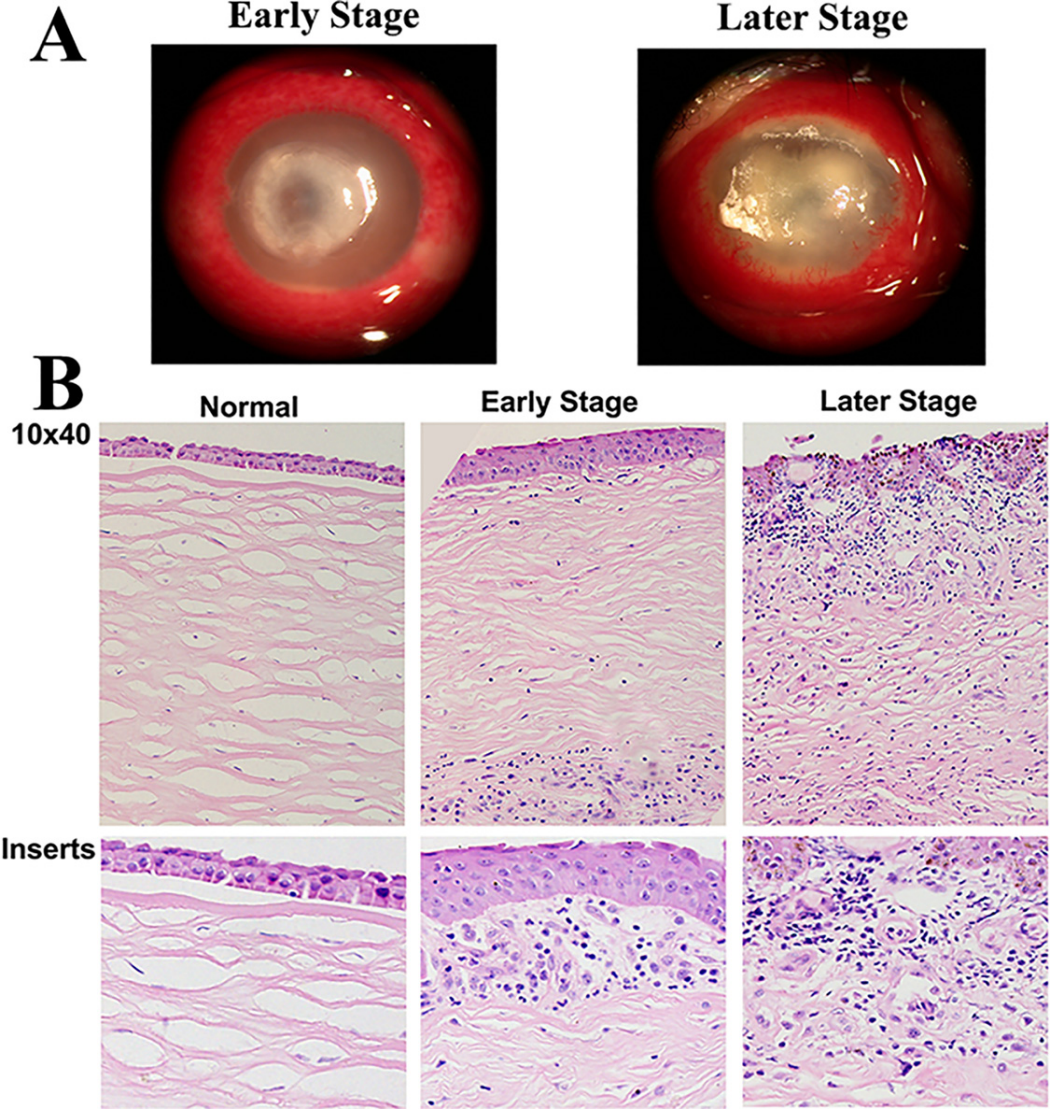
The pathological changes of cornea from patients with fungal keratitis. Slit-lamp photography (A) and HE staining (B) of normal or fungus-infected human corneas in the early and late stage. Magnifications: ×40(low magnification), ×100 (insets).

**Fig 2:**
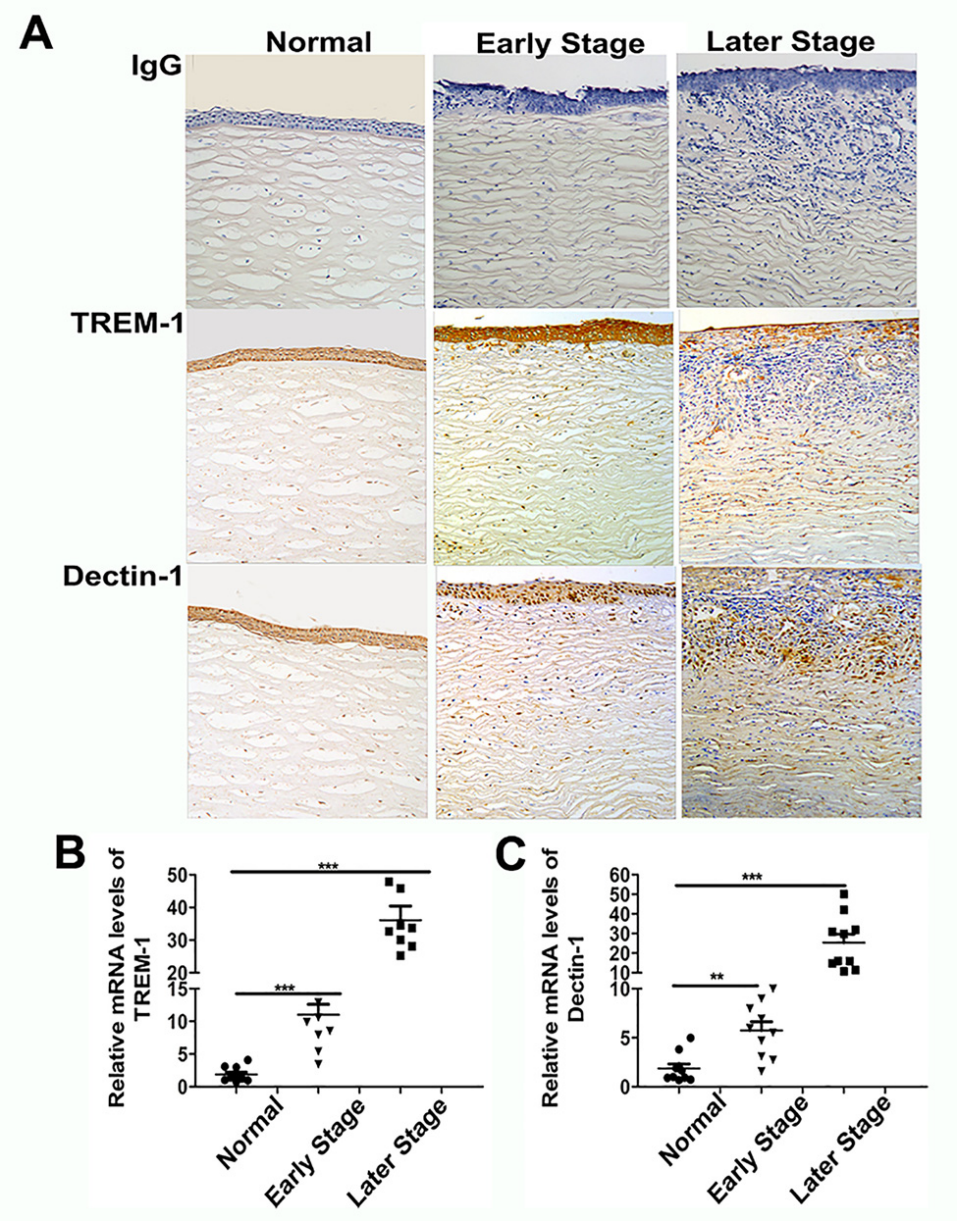
Expressions of TREM-1 and Dectin-1 in fungus-infected human corneas. TREM-1 and Dectin-1 mRNA levels (A) and distribution patterns of TREM-1 and Dectin-1 (B) were examined both in normal and fungus-infected human corneas. Data are presented as mean ± SEM with 10 patients per group.Magnifications: ×20. *, p<0.05; **, p<0.01; ***, p<0.001.

To explore whether TREM-1 and Dectin-1 participate in fungal keratitis, fungus-infected corneas were collected to test expressions of TREM-1 and Dectin-1. Slit-lamp photography showed obvious stromal infiltration and serious corneal ulcers in fungus-infected corneas (Fig 1A). Histological analysis revealed that compared with corneas in the early stage, increased edema was noted in fungus-infected corneas in the late stage, and more infiltrating inflammatory cells accumulated in the corneal stroma over the course of fungal invasion (Fig 1B). Immunohistochemistry revealed increasing-positive staining for TREM-1 and Dectin-1 in the corneal epithelium and stroma from the early to the later stage (Fig 2A). The real-time PCR data (Fig 2B) showed that the expressions of TREM-1 and Dectin-1 were significantly increased in fungus-infected corneas. When compared with normal controls, TREM-1 expression levels were approximately 12-fold higher in the early stage and 40-fold higher in the late stage of fungal keratitis (both *P*<0.001), while Dectin-1 expression levels were 8-fold higher in the early stage and 30-fold higher in the late stage (*P*<0.01 and *P*<0.001, respectively). These results indicate that both TREM-1 and Dectin-1 expression levels were dynamically correlated with the progression of fungal keratitis.

### Characterization of the Cellular Infiltrate in fungus-infected human corneas

**Fig 3:**
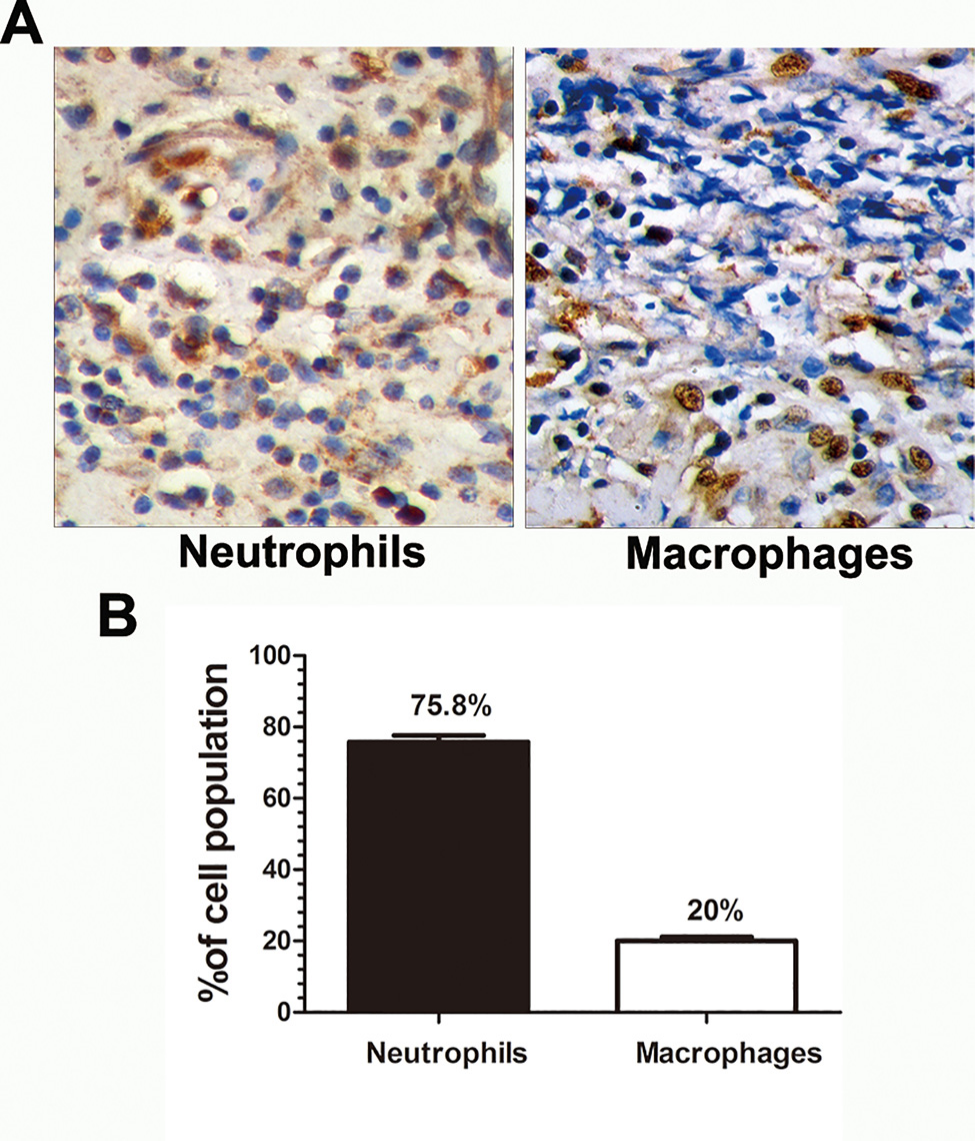
Cellular composition of fungus-infected human corneas. Representative pictures showed the cellular infiltrate in fungus-infected human corneas by immunostaining with antibodies to neutrophils and macrophages (A). The percentages of neutrophils and macrophages were determined by counting 100 cells from 10 patients of fungal keratitis. Magnifications: ×100.

To examine the infiltrated inflammatory cells in fungus-infected human corneas, paraffin sections were immunostained with Gr-1 Ab and anti-macrophage Ab to assess the quantity and morphology of neutrophils and macrophages. We found that the average percentage of neutrophils was 75.8% and macrophages was 20% (Fig 3B); and representative images were also shown in which positive staining were depicted as brown dots (Fig 3A).

### Expressions of TREM-1 and Dectin-1 in the murine fungal keratitis model

**Fig 4:**
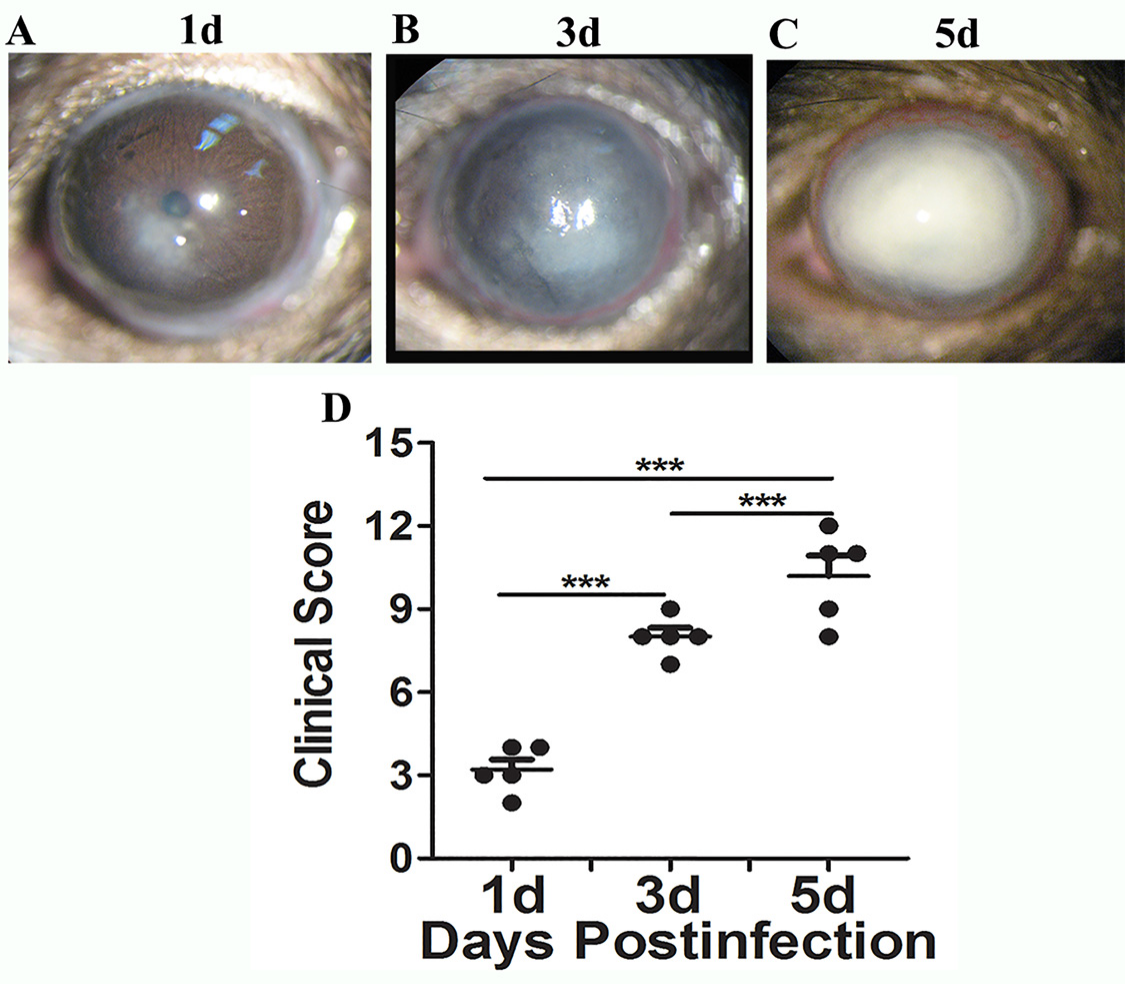
Corneal images and clinical score in the murine fungal keratitis model. C57BL/6 (B6) mice were infected with *Aspergillus fumigatus* following routine protocols. Corneal images (A,B,C) and clinical scores (D) indicated the characteristics of corneal lesions at 1, 3 and 5 days p.i. Magnification: ×16.

**Fig 5:**
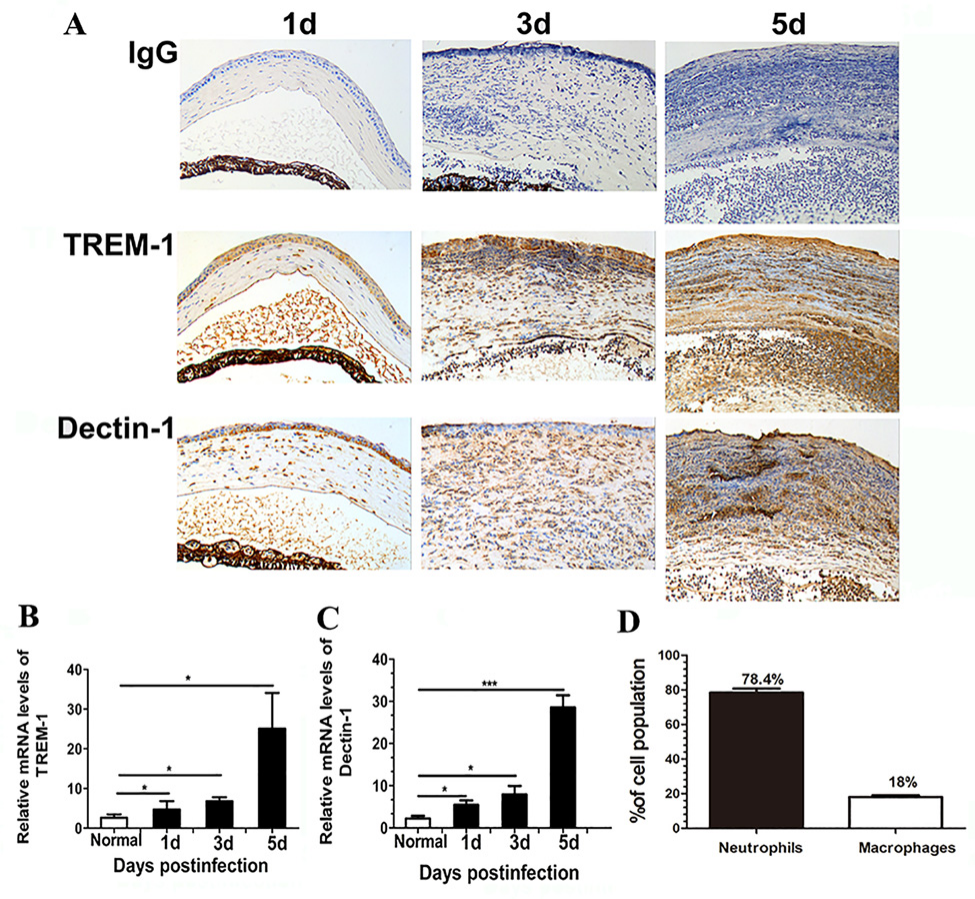
Expressions of TREM-1 and Dectin-1 and Cellular composition in the murine fungal keratitis model. TREM-1 and Dectin-1 protein expression levels were determined using immunohistochemistry in the same samples at the corresponding time points (A). TREM-1 (B) and Dectin-1 (C) mRNA levels were examined in normal (N) and infected B6 mouse corneas at 1, 3 and 5 days p.i.. The percentages of neutrophils and macrophages (D) were determined by counting 100 cells from 5 samples. Magnifications: ×40. *, p<0.05; **, p<0.01; ***, p<0.001.

To further determine the role of TREM-1 and Dectin-1 in fungal keratitis, a well-characterized and accepted murine model of *Aspergillus fumigatus* keratitis was established to mimic human ocular infection. Similar to human fungal keratitis, slit-lamp photograph of corneas showed gradually advanced corneal swelling, corneal ulcers and increased corneal infiltration from day 1 to day 5 p.i. (Fig 4A, 4B and 4C). The clinical score data (Fig 4D) illustrated that the corneal disease progressed gradually at 1, 3, and 5 days p.i., with average scores of 3, 7, and 10, respectively (all *P*<0.001). Additionally, immunohistochemistry revealed significant positive staining for TREM-1 and Dectin-1 (depicted as brown dots) in the corneal epithelium and stroma from 1 to 5 days p.i. (Fig 5A). No staining was observed in the isotype-matched mouse IgG controls in both normal and infected corneas. The mRNA levels and distribution patterns of TREM-1 and Dectin-1 in fungus-infected corneas were examined by real-time PCR and immunohistochemistry, respectively. The real-time PCR data showed that the mRNA levels of TREM-1 and Dectin-1 were significantly upregulated in a time-dependent manner and peaked at 5 days p.i. (Fig 5B and 5C, all *P*<0.05). Neutrophils comprised 78.4% averagely of infiltrating cells, with macrophages of 18% (Fig 5D). The results showed that the expression levels of TREM-1 and Dectin-1 and the percentage of inflammatory cells in Mouse were matched with the Human’s.

### Blockade of TREM-1 and Dectin-1 promoted host resistance to infection

**Fig 6:**
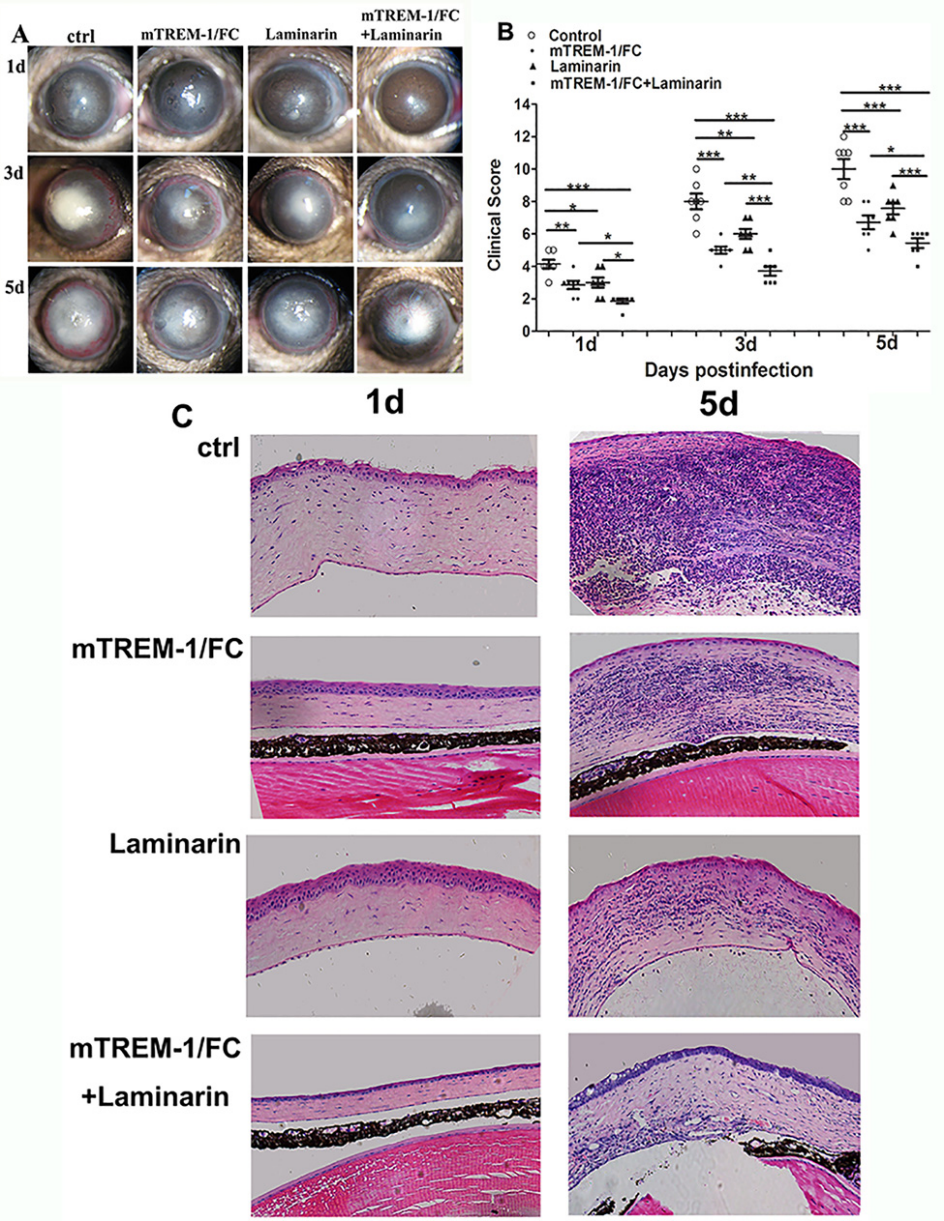
Blockade of TREM-1 and Dectin-1 promoted host resistance to fungal infection. B6 mice were subconjunctivally injected with mTREM-1/IgG fusion protein (1 μg per cornea), soluble β-glucan antagonist laminarin (1 μg per cornea) or both into the right eyes before *Aspergillus fumigatus* infection, followed with administration with the same dosage on each day p.i.. Images of fungus-infected corneas (A) were acquired by slit-lamp photography and corneal scores (B) were recorded at 1, 3 and 5 days p.i. The histopathology of fungus-infected mice corneas was analyzed with HE staining at 5 d p.i. (C). Magnification: ×40. *, p<0.05; **, p<0.01; ***, p<0.001.

Since both TREM-1 and Dectin-1 (mRNA and protein) expressions were significantly up-regulated in B6 corneas after fungal infection, the next series of in vivo studies were designed to determine whether blockade of TREM-1 and Dectin-1 promotes host resistance to fungal infection. mTREM-1/IgG fusion protein and soluble β-glucan antagonist laminarin were subconjunctivally injected to block TREM-1 and Dectin-1, respectively. Slit-lamp photographs revealed that blockade either TREM-1 or Dectin-1 reduced disease severity of fungal kereatits (Fig 6A). Simultaneous inhibition of TREM-1 and Dectin-1 showed synergic protection from fungal infection (Fig 6A). The clinical scores were highest in control group and lowest in TREM-1 and Dectin-1 inhibition group at 1, 3 and 5 days p.i. (Fig 6B, all *P*<0.05).

Furthermore, HE staining (Fig 6C) confirmed that infected corneas of control group exhibited the most serious stromal swelling, with many inflammatory cells infiltrating the stroma and anterior chamber, while inhibition of TREM-1 or Dection-1 reduced corneal disease (Fig 6C). When both TREM-1 and Dection-1 were blocked, corneas showed a relatively intact epithelium with slight corneal infiltration (Fig 6C).

### Blockade of TREM-1 and Dectin-1 decreased Th1 responses, but increased Th2 responses

**Fig 7:**
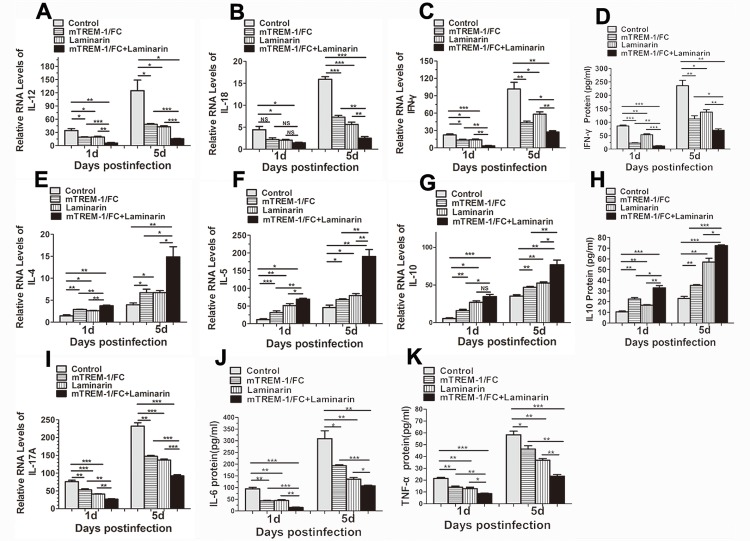
Th1, Th2 and pro-inflammatory cytokines production in fungus-infected cornea after blocking TREM-1 and Dectin-1. B6 mice were subconjunctivally injected with mTREM-1/IgG fusion protein (1 μg per cornea), soluble β-glucan antagonist laminarin (1 μg per cornea) or both into the right eyes before *Aspergillus fumigatus* infection, followed with administration with the same dosage on each day p.i.. The mRNA expression levels of Th1-type cytokines, including IL-12 (A), IL-18 (B) and IFN-γ (C), Th2 cytokines, including IL-4 (E), IL-5 (F) and IL-10 (G), and IL-17A(I) were detected with real-time PCR at 1 and 5 days p.i. The protein levels of IFN-γ (D), IL-10 (H), IL-6 (J) and TNF-α (K) were determined with ELISA at 1 and 5 days p.i. in each group. Data are the mean ± SEM and represent individual experiments, each including 5 animals/group/time. *, p<0.05; **, p<0.01; ***, p<0.001.

To explore the mechanism by which TREM-1 and Dectin-1 modulates the immune response, mRNA levels of selected Th1 and Th2 cytokines were analyzed by real-time PCR in infected corneas of mTREM-1/Fc, laminarin or control-treated B6 mice at 1 and 5 days p.i.. At different time points, Compared with controls, blockade of mTREM-1 or Dectin-1 significantly down-regulated the levels of Th1 cytokines including IL-12 (Fig 7A, all *P*<0.05), IL-18 (Fig 7B, all *P*<0.05) and IFN-γ (Fig 7C, all *P*<0.05), but increased Th2 cytokines, including IL-4 (Fig 7E, all *P*<0.05), IL-5 (Fig 7F, all *P*<0.05) and IL-10 (Fig 7G, all *P*<0.05). Additionally, ELISA data showed that inhibiting TREM-1 and Dectin-1 simultaneously decreased IFN-γ protein levels (Fig 7D, all *P*<0.05) and increased in IL-10 protein levels (Fig 7H, all *P*<0.05). Moreover, RNA expression levels of IL-17A (Fig 7I, all P<0.05) and protein expression levels of IL-6 (Fig 7J, all *P*<0.05) and TNF-α (Fig 7K, all *P*<0.05) decreased after TREM-1 or Dectin-1 inhibition at 1 and 5 days p.i..

## Discussion

The histopathology of fungal keratitis correlates closely with the immune response [16]. Recognition of fungi initiates a variety of inflammatory events in the cornea, such as infiltration of inflammatory cells as well as the production of inflammatory cytokines, chemokines and other soluble mediators. These factors initiate inflammatory responses and help shape the development of antifungal immunity [16, 17]. In fungal keratitis, moderate inflammation promotes fungal clearance and induces healing of injured tissue. However, if the process is not controlled properly, excessive host inflammatory response will be aggravate tissue damage, and result in corneal perforation ultimately [18]. In this study, we find that blockage of TREM-1 and Dectin-1 can alleviate fungal keratitis by modulating Th1/Th2 immune responses.

Triggering receptor in myeloid cells-1 (TREM-1) is reported to be a critical inflammation-amplifier and has recently been implicated as a key molecule in the regulation of innate immune responses [19]. TREM-1 is found on neutrophils, monocytes and macrophages [11, 19–21] and its expression is increased by the presence of LPS, bacteria or fungi [19–21]. TREM-1 expression has been reported in *Aspergillus*-containing granulomas, suggesting that this receptor might be involved in the antifungal responses of the host.[11] Further study indicated that TREM-1 regulates immune responses to *A*. *fumigatus* during fungal asthma in fungus-infected lungs, clearly revealing a role for TREM-1 in fungal infection [10]. In this study, we first demonstrated that the mRNA level of TREM-1 was significantly increased and the majority of infiltrated cells were neutrophils,followed by macrophages in corneas after fungal infection. Moreover, the increased expression levels were closely associated with the pathological process and severity of corneal damage.

The specific ligand for TREM-1 remains unknown, however, once activated, TREM-1 acts as a critical amplifier of inflammatory signaling[22]. Treatment of inflammatory cells with TREM-1 ligation in vitro induces secretion of pro-inflammatory cytokines and chemokines, immediate degranulation, respiratory burst and phagocytosis [23], [24]. Our in vivo studies showed that a blockade of TREM-1 alleviated disease progression. However, a few infected corneas still became perforated in the late stage. Thus, it is possible that other key molecules aside from TREM-1 act simultaneously to mediate pathogenesis in fungal keratitis.

Dectin-1 which is widely expressed on neutrophils, macrophages and dendritic cells, recognizes β-glucans and is critical for the recognition and phagocytosis of fungi [8]. Our study is consistent with recent reports [8], showing that the expression of Dectin-1 was elevated in fungal keratitis, which were mainly infiltrated with neutrophils and macrophages in human and mouse fungi-infected cornea[25]. The expression of Dectin-1 in fungi-infected corneas was enhanced in the early stage and subsequently changed with the progression of corneal damage in fungal keratitis. Notably, the increase of Dectin-1 was correlated with that of TREM-1. Interestingly, Leal and Goodridge [26] indicated that TLRs and Dectin-1 work together to detect invading fungi, while TREM-1 also acts synergistically with TLRs to modulate inflammatory responses.[19]. Taken together, these findings indicate the close relationship among PRRs. Based on these studies, we hypothesized that TREM-1 and Dectin-1 may show similar increasing trends and act synergistically in fungal keratitis.

In a mouse model of fungal keratitis, we blocked TREM-1 and Dectin-1 simultaneously and observed that the clinical scores were lower than those in other groups in which TREM-1 or Dectin-1 was inhibited alone. Additionally, the infiltration of inflammatory cells in the cornea was obviously alleviated. These phenomena implied that TREM-1 and Dectin-1 may synergistically stimulate the inflammatory response. It is possible that fungal infection-triggered TREM-1 signaling activates downstream spleen tyrosine kinase (Syk) and caspase-recruitment domain 9 (CARD9) [8, 19], which is also necessary for Dectin-1 signaling activation. TREM-1 and Dectin-1 signaling converge on Syk and CARD9 and act synergically to amplify inflammatory response. This may explain why blocking TREM-1 and Dectin-1 together can alleviate fungal keratitis more effectively.

According to previous studies, fungal keratitis can cause dysregulation of the immune system following fungal invasion, and it is important to maintain a balance between inflammatory and anti-inflammatory responses [27]. Continuous release of Th1 cytokines induced by fungal antigen from activated neutrophils, macrophages, dendritic cells,ect, may cause excessive inflammation and accelerate tissue destruction. Meanwhile, Th2 cytokines from autocrine of Th2 cells or activated neutrophils, macrophages and keratinocytes attenuate the immune-related inflammation and alleviate the severity of corneal lesions (9, 29). Th2 type cytokine has been reported to be anti-inflammatory cytokines in fungal infection and allergic immune response [28, 29], because they could downregulate the inflammatory cells’ activation. In Fig 6C, it is easy to observe from the HE staining that blocking TREM-1and Dectin-1 could obviously reduce the amounts of infiltrated inflammatory cells, including neutrophils and macrophages. As we all known, neutrophils and macrophages played important role on amplifying the fungi-induced inflammatory response, and the upregulation of Th2 response after blocking TREM-1 and Dectin-1 reduced the infiltration and suppressed the activation of inflammatory cells, therefore they benefited to alleviate the fungal infection. Compared with the inhibition of either TREM-1 or Dectin-1 alone, we found that inhibiting both TREM-1 and Dectin-1 group infiltrated with the least inflammatory cells, then decreased the expression of Th1 cytokines (IL-12, IL-18 and IFN-γ), and increased the expression of Th2 cytokines (IL-4, IL-5 and IL-10).

Additionally, our study illustrated that the productions of pro-inflammatory cytokines including IL-17A, IL-6 and TNF-ɑ were dramatically decreased by inhibiting TREM-1 and Dectin-1; IL-17A was released by Th17 cells, and IL-6 and TNF-ɑ were from the activated the neutrophils and macrophages, which played central role on resisting fungal invasion [30, 31]. Thus, the reduced neutrophils and macrophages infiltrations after inhibition were responsible for the decreased proinflammatory cytokines in diseased corneas. Conclusively, blockade of TREM-1 and Dectin-1 suppressed the activation of proinflammatory cytokines and the secretion of Th1/Th2 inflammatory cytokines, preventing excessive inflammatory responses as well as cellular infiltrates and therefore ameliorating the pathological damage of infected corneas.

To summarize, this study firstly demonstrated that blockage of TREM-1 and Dectin-1 can reduce the severity of corneal damage in a mouse model of fungal keratitis by downregulating the inflammatory response. Our study provides a better understanding of the pathologic mechanism of the inflammatory cytokine network in fungal keratitis. Furthermore, inhibiting the expression of TREM-1 and Dectin-1 can exert anti-inflammatory effects and has promising potential for treatment of fungal keratitis.

## References

[1] Thomas PA, Kaliamurthy J. Mycotic keratitis: epidemiology, diagnosis and management. Clin Microbiol Infect. 2013;19(3):210–20. Epub 2013/02/13. 10.1111/1469-0691.12126 . 23398543

[2] Chang DC, Grant GB, O'Donnell K, Wannemuehler KA, Noble-Wang J, Rao CY, et al Multistate outbreak of Fusarium keratitis associated with use of a contact lens solution. JAMA. 2006;296(8):953–63. Epub 2006/08/24. 10.1001/jama.296.8.953 . 16926355

[3] Keay LJ, Gower EW, Iovieno A, Oechsler RA, Alfonso EC, Matoba A, et al Clinical and microbiological characteristics of fungal keratitis in the United States, 2001–2007: a multicenter study. Ophthalmology. 2011;118(5):920–6. Epub 2011/02/08. 10.1016/j.ophtha.2010.09.011 21295857 PMC3673009

[4] Prajna NV, Krishnan T, Mascarenhas J, Srinivasan M, Oldenburg CE, Toutain-Kidd CM, et al Predictors of outcome in fungal keratitis. Eye (Lond). 2012;26(9):1226–31. 10.1038/eye.2012.99 22744392 PMC3443844

[5] Gordon S. Pattern recognition receptors: doubling up for the innate immune response. Cell. 2002;111(7):927–30. Epub 2003/01/01. . 12507420 10.1016/s0092-8674(02)01201-1

[6] Osorio F, Reis e, Sousa C. Myeloid C-type lectin receptors in pathogen recognition and host defense. Immunity. 2011;34(5):651–64. Epub 2011/05/28. 10.1016/j.immuni.2011.05.001 . 21616435

[7] Leal SM Jr, Cowden S, Hsia YC, Ghannoum MA, Momany M, Pearlman E. Distinct roles for Dectin-1 and TLR4 in the pathogenesis of Aspergillus fumigatus keratitis. PLoS Pathog. 2010;6:e1000976 Epub 2010/07/10. 10.1371/journal.ppat.1000976 20617171 PMC2895653

[8] Dennehy KM, Brown GD. The role of the beta-glucan receptor Dectin-1 in control of fungal infection. J Leukoc Biol. 2007;82(2):253–8. Epub 2007/05/04. 10.1189/jlb.1206753 . 17475782

[9] Yuan X, Wilhelmus KR. Toll-like receptors involved in the pathogenesis of experimental Candida albicans keratitis. Invest Ophthalmol Vis Sci. 2010;51(4):2094–100. Epub 2009/11/26. 10.1167/iovs.09-4330 19933194 PMC2868407

[10] Wu M, Peng A, Sun M, Deng Q, Hazlett LD, Yuan J, et al TREM-1 amplifies corneal inflammation after Pseudomonas aeruginosa infection by modulating Toll-like receptor signaling and Th1/Th2-type immune responses. Infection and immunity. 2011;79(7):2709–16. Epub 2011/05/11. 10.1128/IAI.00144-11 21555403 PMC3191966

[11] Bouchon A, Facchetti F, Weigand MA, Colonna M. TREM-1 amplifies inflammation and is a crucial mediator of septic shock. Nature. 2001;410(6832):1103–7. Epub 2001/04/27. 10.1038/35074114 . 11323674

[12] van Bremen T, Dromann D, Luitjens K, Dodt C, Dalhoff K, Goldmann T, et al Triggering receptor expressed on myeloid cells-1 (Trem-1) on blood neutrophils is associated with cytokine inducibility in human E. coli sepsis. Diagn Pathol. 2013;8:24 10.1186/1746-1596-8-24 23414215 PMC3584978

[13] Wu TG, Wilhelmus KR, Mitchell BM. Experimental keratomycosis in a mouse model. Invest Ophthalmol Vis Sci. 2003;44(1):210–6. Epub 2002/12/31. . 12506077 10.1167/iovs.02-0446

[14] Huang H, Ostroff GR, Lee CK, Agarwal S, Ram S, Rice PA, et al Relative contributions of dectin-1 and complement to immune responses to particulate beta-glucans. Journal of immunology. 2012;189(1):312–7. Epub 2012/06/01. 10.4049/jimmunol.1200603 22649195 PMC3381926

[15] Gantner BN, Simmons RM, Canavera SJ, Akira S, Underhill DM. Collaborative induction of inflammatory responses by dectin-1 and Toll-like receptor 2. J Exp Med. 2003;197(9):1107–17. Epub 2003/04/30. 10.1084/jem.20021787 12719479 PMC2193968

[16] Garcia-Vidal C, Viasus D, Carratala J. Pathogenesis of invasive fungal infections. Curr Opin Infect Dis. 2013;26(3):270–6. Epub 2013/03/02. 10.1097/QCO.0b013e32835fb920 . 23449139

[17] Leal SM Jr, Pearlman E. The role of cytokines and pathogen recognition molecules in fungal keratitis—Insights from human disease and animal models. Cytokine. 2012;58(1):107–11. 10.1016/j.cyto.2011.12.022 22280957 PMC3290702

[18] Brown GD. Innate antifungal immunity: the key role of phagocytes. Annu Rev Immunol. 2011;29:1–21. Epub 2010/10/13. 10.1146/annurev-immunol-030409-101229 20936972 PMC3434799

[19] Arts RJ, Joosten LA, van der Meer JW, Netea MG. TREM-1: intracellular signaling pathways and interaction with pattern recognition receptors. J Leukoc Biol. 2013;93(2):209–15. Epub 2012/10/31. 10.1189/jlb.0312145 . 23108097

[20] Buckland KF, Ramaprakash H, Murray LA, Carpenter KJ, Choi ES, Kunkel SL, et al Triggering receptor expressed on myeloid cells-1 (TREM-1) modulates immune responses to Aspergillus fumigatus during fungal asthma in mice. Immunol Invest. 2011;40(7–8):692–722. Epub 2011/05/20. 10.3109/08820139.2011.578270 . 21592044 PMC5540193

[21] McClellan SA, Huang X, Barrett RP, Lighvani S, Zhang Y, Richiert D, et al Matrix metalloproteinase-9 amplifies the immune response to Pseudomonas aeruginosa corneal infection. Invest Ophthalmol Vis Sci. 2006;47(1):256–64. Epub 2005/12/31. 10.1167/iovs.05-1050 . 16384971

[22] Sharif O, Knapp S. From expression to signaling: roles of TREM-1 and TREM-2 in innate immunity and bacterial infection. Immunobiology. 2008;213(9–10):701–13. Epub 2008/10/18. 10.1016/j.imbio.2008.07.008 . 18926286

[23] Tessarz AS, Weiler S, Zanzinger K, Angelisova P, Horejsi V, Cerwenka A. Non-T cell activation linker (NTAL) negatively regulates TREM-1/DAP12-induced inflammatory cytokine production in myeloid cells. Journal of immunology. 2007;178(4):1991–9. . 17277102 10.4049/jimmunol.178.4.1991

[24] Ornatowska M, Azim AC, Wang X, Christman JW, Xiao L, Joo M, et al Functional genomics of silencing TREM-1 on TLR4 signaling in macrophages. Am J Physiol Lung Cell Mol Physiol. 2007;293(6):L1377–84. 10.1152/ajplung.00140.2007 17905855 PMC3969455

[25] Karthikeyan RS, Leal SM Jr, Prajna NV, Dharmalingam K, Geiser DM, Pearlman E, et al Expression of innate and adaptive immune mediators in human corneal tissue infected with Aspergillus or fusarium. The Journal of infectious diseases. 2011;204(6):942–50. 10.1093/infdis/jir426 21828275 PMC3156922

[26] Goodridge HS, Underhill DM. Fungal Recognition by TLR2 and Dectin-1. Handb Exp Pharmacol. 2008;(183):87–109. Epub 2007/12/12. 10.1007/978-3-540-72167-3_5 . 18071656

[27] Jurkunas U, Behlau I, Colby K. Fungal keratitis: changing pathogens and risk factors. Cornea. 2009;28(6):638–43. Epub 2009/06/11. 10.1097/ICO.0b013e318191695b . 19512908

[28] O'Dea EM, Amarsaikhan N, Li H, Downey J, Steele E, Van Dyken SJ, et al Eosinophils are recruited in response to chitin exposure and enhance Th2-mediated immune pathology in Aspergillus fumigatus infection. Infection and immunity. 2014;82(8):3199–205. 10.1128/IAI.01990-14 24842927 PMC4136210

[29] Schulze-Koops H, Kalden JR. The balance of Th1/Th2 cytokines in rheumatoid arthritis. Best Pract Res Clin Rheumatol. 2001;15(5):677–91. Epub 2002/01/29. 10.1053/berh.2001.0187 . 11812015

[30] Zhang H, Li H, Li Y, Zou Y, Dong X, Song W, et al IL-17 plays a central role in initiating experimental Candida albicans infection in mouse corneas. European journal of immunology. 2013;43(10):2671–82. 10.1002/eji.201242891 . 23843112

[31] Taylor PR, Leal SM Jr, Sun Y, Pearlman E. Aspergillus and Fusarium corneal infections are regulated by Th17 cells and IL-17-producing neutrophils. Journal of immunology. 2014;192(7):3319–27. 10.4049/jimmunol.1302235 24591369 PMC4020181

